# Small molecule annotation for the Protein Data Bank

**DOI:** 10.1093/database/bau116

**Published:** 2014-11-23

**Authors:** Sanchayita Sen, Jasmine Young, John M. Berrisford, Minyu Chen, Matthew J. Conroy, Shuchismita Dutta, Luigi Di Costanzo, Guanghua Gao, Sutapa Ghosh, Brian P. Hudson, Reiko Igarashi, Yumiko Kengaku, Yuhe Liang, Ezra Peisach, Irina Persikova, Abhik Mukhopadhyay, Buvaneswari Coimbatore Narayanan, Gaurav Sahni, Junko Sato, Monica Sekharan, Chenghua Shao, Lihua Tan, Marina A. Zhuravleva

**Affiliations:** ^1^Protein Data Bank in Europe (PDBe), EMBL-EBI, Wellcome Trust Genome Campus, Hinxton CB10 1SD, UK, ^2^RCSB Protein Data Bank (RCSB PDB), Department of Chemistry and Chemical Biology, Rutgers University, Piscataway, NJ 08854-8087, USA and ^3^Protein Data Bank Japan (PDBj), Osaka University, Osaka, Japan

## Abstract

The Protein Data Bank (PDB) is the single global repository for three-dimensional structures of biological macromolecules and their complexes, and its more than 100 000 structures contain more than 20 000 distinct ligands or small molecules bound to proteins and nucleic acids. Information about these small molecules and their interactions with proteins and nucleic acids is crucial for our understanding of biochemical processes and vital for structure-based drug design. Small molecules present in a deposited structure may be attached to a polymer or may occur as a separate, non-covalently linked ligand. During curation of a newly deposited structure by wwPDB annotation staff, each molecule is cross-referenced to the PDB Chemical Component Dictionary (CCD). If the molecule is new to the PDB, a dictionary description is created for it. The information about all small molecule components found in the PDB is distributed via the ftp archive as an external reference file. Small molecule annotation in the PDB also includes information about ligand-binding sites and about covalent and other linkages between ligands and macromolecules. During the remediation of the peptide-like antibiotics and inhibitors present in the PDB archive in 2011, it became clear that additional annotation was required for consistent representation of these molecules, which are quite often composed of several sequential subcomponents including modified amino acids and other chemical groups. The connectivity information of the modified amino acids is necessary for correct representation of these biologically interesting molecules. The combined information is made available via a new resource called the Biologically Interesting molecules Reference Dictionary, which is complementary to the CCD and is now routinely used for annotation of peptide-like antibiotics and inhibitors.

## Introduction

The Protein Data Bank (PDB) is the single global repository for three-dimensional (3D) structures of biological macromolecules and their complexes (1). The four partners of the Worldwide PDB organization (wwPDB; http://wwpdb.org) are the Research Collaboratory for Structural Bioinformatics (RCSB PDB; http://rcsb.org) (2), the PDB in Europe (PDBe; http://pdbe.org) (3), the PDB Japan (PDBj; http://pdbj.org) (4) and the Biological Magnetic Resonance Bank (BMRB; http://bmrb.wisc.edu) (5). They act as deposition, curation and distribution centres for PDB data. Although the PDB archive is focussed on macromolecules, a wide variety of small molecules are encountered bound to proteins and nucleic acids. Currently, there are >20 000 distinct kinds of small molecule present in the archive, and they are described in the wwPDB Chemical Component Dictionary (CCD). These compounds include metals, ions, cofactors, fatty acids, carbohydrates, proteinogenic (standard) and modified amino acids and nucleotides, chromophores, antibiotics, inhibitors and various other compounds that may be naturally bound to a macromolecule or acquired during purification or crystallization.

The first step in ligand annotation by wwPDB curators is to identify all the distinct chemical entities that are present in a newly deposited structure, including all polymers and small molecules (6). PDB annotation is a complex scientific process that requires understanding of the interactions between small molecules and macromolecules. Aspects of small molecule annotation include:

1. identifying small molecules in a newly deposited PDB entry that are already present in the CCD;
2. creating definitions for any small molecules that are new to the PDB;
3. geometry and stereochemistry validation;
4. evaluating the fit of the model coordinates to the experimental data;
5. identifying any covalent links with other residues or components;
6. annotation of ligand binding sites and
7. extending or updating the annotation in Biologically Interesting molecules Reference Dictionary (BIRD) for peptide-like inhibitor and antibiotic molecules.

## The wwPDB CCD

The number of structures in the PDB archive has grown from 7 in 1971 to >100 000 in 2014 (7, 8). All these structures are experimentally derived atomistic models of biologically important proteins and nucleic acids from a huge variety of organisms. Many proteins in the PDB have substrates, co-factors, reaction products or analogues of such compounds bound to them. In addition, many proteins and nucleic acids contain modified amino acid or nucleotide residues. Hence, identifying monomeric components incorporated in the polymers and ligands is an important first step of PDB annotation (6).

**Figure 1.**
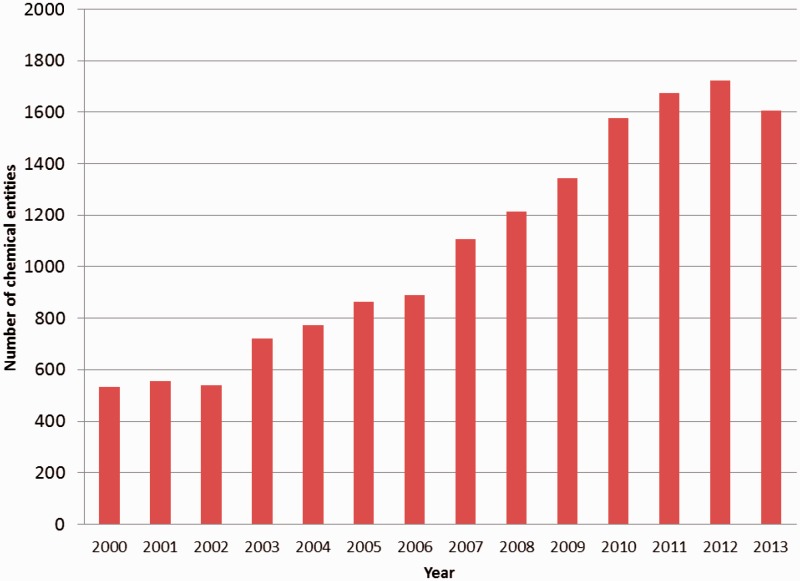
Number of new PDB chemical entity definitions created annually between 2000 and 2013.

The chemical-component annotation of a PDB entry involves identification of every small molecule that is present in the structure, either as part of a polymer or as a non-covalently bound ligand. With the increasing number of structures in the PDB, the number of unique chemical entities associated with them is increasing as well (Figure 1). For annotation purposes it is important to identify and describe the chemical entities that are deposited to the PDB in a systematic and consistent manner. The wwPDB partners achieved this through the creation of a chemical reference dictionary. This contains the description of every unique chemical entity, which can then be reused in subsequent depositions that contain the same entity. This dictionary is known as the wwPDB CCD and currently contains chemical definitions of more than 20 000 distinct chemical entities.

The wwPDB annotation software verifies for all small molecules in a new deposition if they already occur in the CCD. When a small molecule in the deposited entry matches an existing chemical definition in the CCD, it is assigned the same three-character identifier as in the CCD. The atom nomenclature of the small molecule in the entry is updated to reflect the dictionary definition. If a molecule is new to the PDB, then a dictionary description is created for it. The atomic coordinates from the deposited structure are used for the initial deduction of the connectivity, bond orders and stereochemistry of the molecule. In cases where the small molecule has poor geometry, extra annotation is required to define the correct connectivity and bond orders. The dictionary definition for every molecule contains both coordinates generated with idealized geometry and example experimental coordinates along with machine-readable chemical descriptors, such as SMILES strings (9), InChi (10) and InChi keys. The CCD definition also contains the systematic name, synonyms, chemical formula, formula weight and formal charge of the molecule.

**Figure 2.**
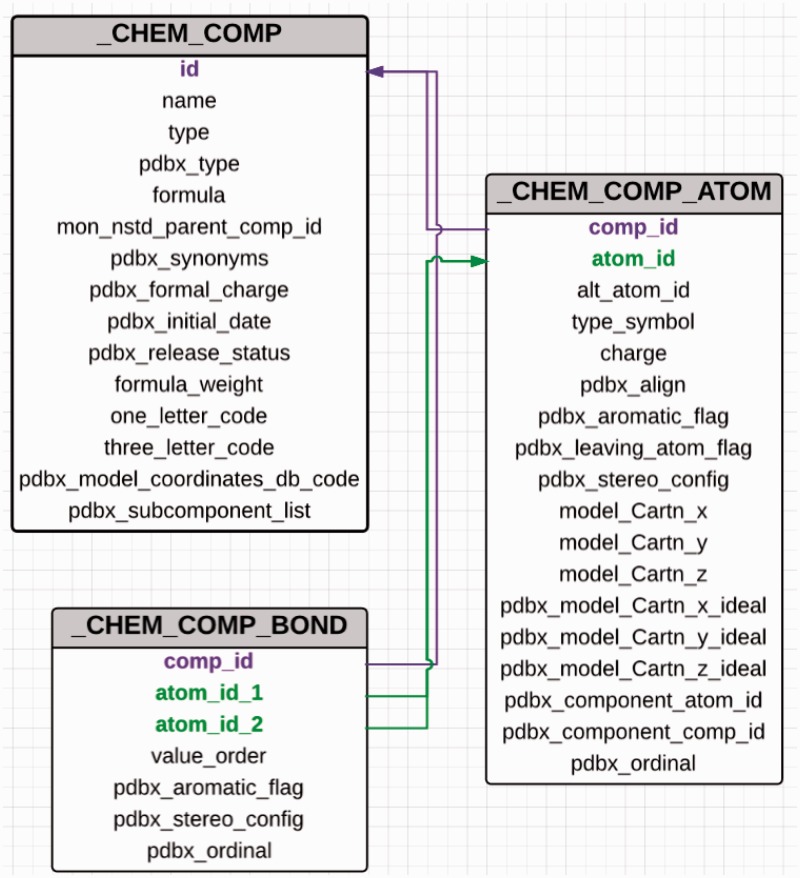
Abbreviated category relationship diagram for the key CIF categories that are used in the CCD. Three major categories _chem_comp, _chem_comp_bond and _chem_comp_atom are joined together to generate the machine readable dictionary description of the chemical entity. The unique three character code assigned to every new chemical entity acts as the primary key in the _chem_comp category.id (coloured in purple) and is used to connect the other categories (_chem_comp_bond.comp_id and _chem_comp_atom.comp_id).

The PDB uses PDB exchange/macromolecular Crystallographic Information File format (PDBx/mmCIF) (11) to describe the contents of a PDB entry. The advantages of using mmCIF as the data model include its flexibility and extensibility, which allow addition of new data items to address the ever-increasing size and complexity of deposited structures. The CCD is also maintained in CIF format (11) and every chemical entity in the dictionary is assigned a unique three-character identifier. Each CCD entry contains five CIF categories that provide the machine-readable chemical description of the small molecule (Figure 2). The unique three-character code assigned to a chemical component is used as the primary key to connect the different categories in the dictionary. The *_chem_comp* category contains the name, synonyms, chemical formula, formula weight and formal charge of the molecule along with some information about the parent PDB entry, which was used to construct the dictionary entry. For entities such as chromophores and modified amino acids and nucleic acids, the data item*_chem_comp.mon_nstd_parent_comp_id* provides information about the ‘parent’ of the compound. For example, the amino acid phosphotyrosine is derived from tyrosine, which is therefore its parent. The chromophore CRO present in green fluorescent protein is derived from the tripeptide Ser-Tyr-Gly. Hence, the dictionary definition of CRO has Ser-Tyr-Gly listed as the parent of this chromophore. The _*chem_comp* category provides structural classification information about the compound. The data items *_chem_ comp.type* and *_chem_comp.pdbx_type* are used to annotate the type and class of the molecule, respectively. A standard or a sidechain modified l-amino acid is a member of the class ‘L-peptide linking’. Amino acids and their modified forms occur in proteins and are designated as type ‘ATOMP’. Sugars can be classified as ‘D-saccharide’ or ‘L-saccharide’ depending on their configuration and they are annotated as type ‘ATOMS’.

The *_chem_comp_atom* category contains atom-level details about the compound. This category not only includes atom name, element type, aromaticity flag, chirality and charge for every atom but also both the generated and example experimental 3D coordinates for the entire chemical entity. Chemistry software CORINA (12) is used to produce the programmatically generated conformation. The bond-order information is stored in the *_chem_ comp_bond* category, whereas the SMILES and InChi strings are in the *_pdbx_chem_comp_descriptor* category. Standard chemistry software such as from ACDlabs (http://www.acdlabs.com), CACTVS (13) and OpenEye (14) is used to generate the systematic name, SMILES and InChI descriptors for the compound. As part of a wwPDB collaboration with the Cambridge Crystallography Data Centre, Cambridge Structural Database (CSD) coordinates for those small molecules in the PDB that also exist in CSD will be included in the future as well.

**Figure 3.**
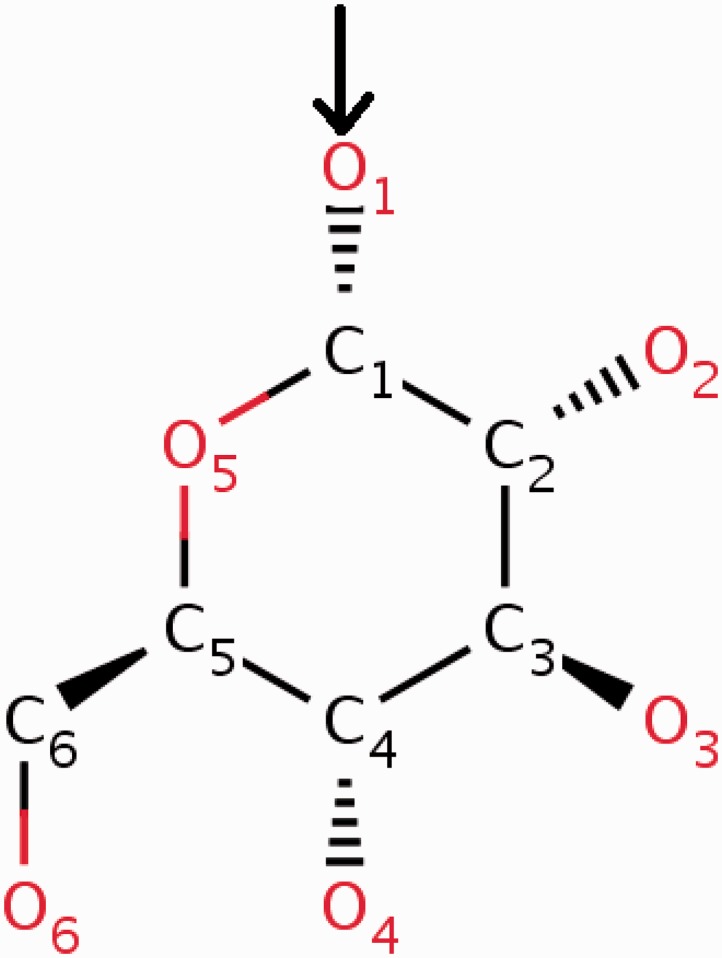
α-d-Glucose can form α(1–4) glycosidic linkages with other carbohydrate molecules. During the oligomerization process, the O1 oxygen (highlighted in the figure) of the glucose is eliminated by the O4 oxygen of the other carbohydrate. To account for this condensation reaction, the O1 oxygen of α-d-glucose (GLC) is annotated in the CCD as a leaving atom. The two-dimensional diagram in this figure is a copy of the image from the RCSB PDB website (http://rcsb.org/pdb/ligand/ligandsummary.do?hetId=GLC). It was generated using the ChemAxon software (http://www.chemaxon.com).

**Figure 4.**
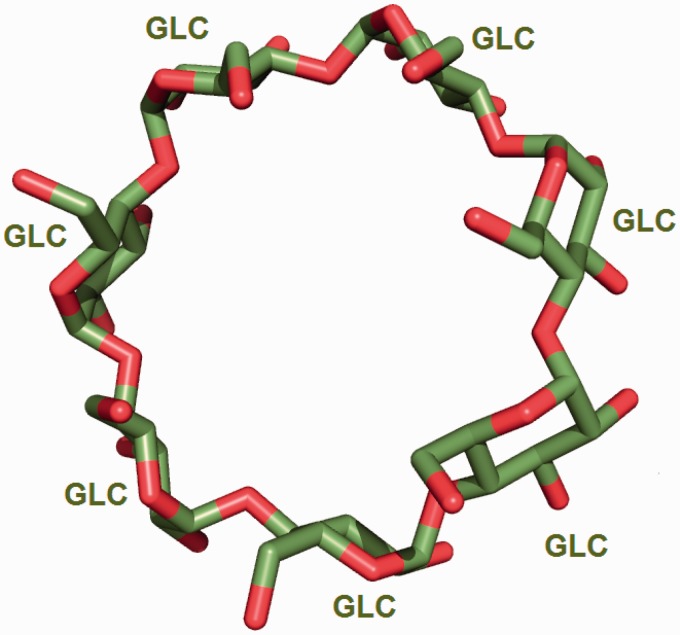
Seven α-d-glucose (GLC) molecules undergo condensation reaction to form the circular oligosaccharide β-cyclodextrin [from PDB entry 2v8l (30)].

The wwPDB annotation guidelines require the CCD definition to pertain to the neutral unbound form of every compound. In cases where a new chemical entity is covalently linked to another small molecule or to a polymer, a leaving atom (usually an oxygen atom labelled as OXT) is introduced in the chemical definition of the compound. In this way the newly built chemical definition can be reused in future depositions where the same compound may occur either in covalently bound form or as a ‘free’ ligand. The use of leaving atoms to capture multiple chemical forms of the same compound is especially common for amino acids, nucleotides and carbohydrates (Figures 3 and 4).

## Annotation of ligand-binding sites

Covalently linked ligands are quite common in the PDB, including co-factors such as pyridoxal phosphate and carbohydrates such as *N*-acetylglucosamine. Non-covalently linked ligands are usually bound to one or more polymers through H-bonding, ionic and van der Waals interactions. Some ligands are instrumental to the biological function of a biomacromolecule or complex. Obviously, the binding environment of a ligand is important to stabilize a particular conformational state that facilitates its biological function.

**Figure 5.**
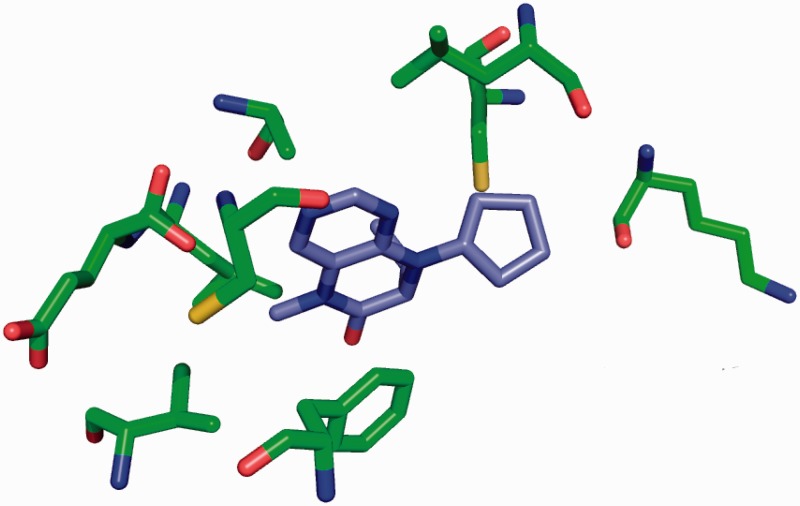
Binding site for the Plk-2 inhibitor (7R)-8-cyclopentyl-7-ethyl-5-methyl-7,8-dihydropteridin-6(5H)-one (3 letter code 11 G) in PDB entry 4i6b (31). The figure depicts the neighbouring residues that are within 3.7 Å of the ligand 11 G.

**Figure 6.**
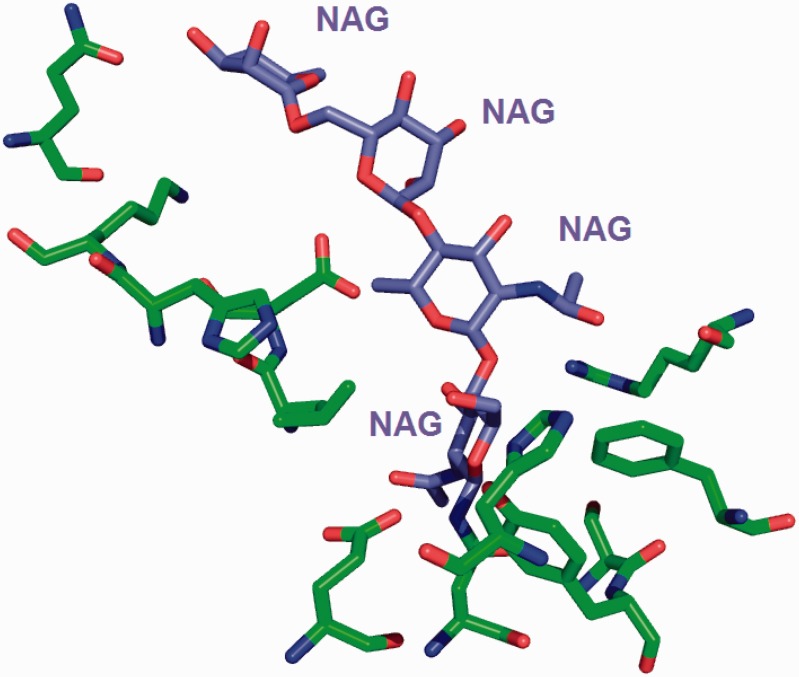
The environment for the oligosaccharide poly-*N*-acetylglucosamine (PNAG) is annotated instead of listing the environment of individual sugars. This avoids repeating the same sugar molecule in multiple binding sites.

**Figure 7.**
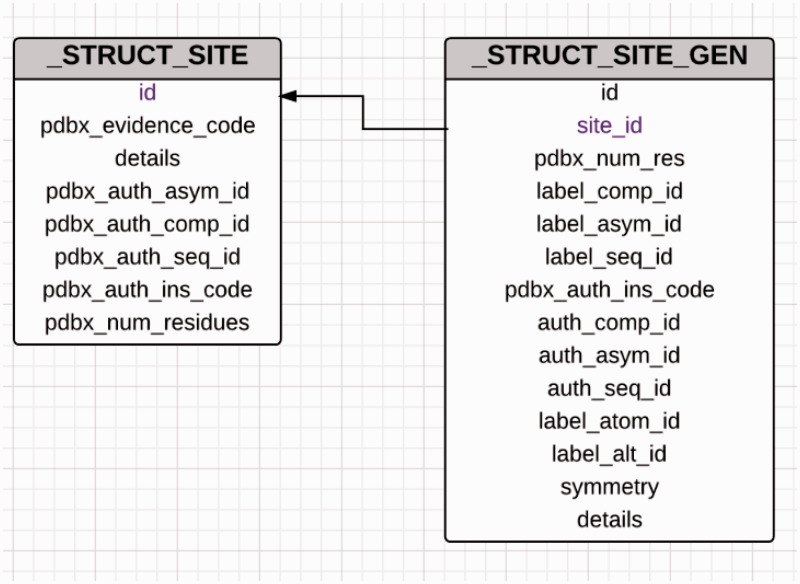
Diagram showing the relationship between the*_struct_site* and _struct_site_gen categories used for annotation of ligand-binding sites. The _struct_site category holds information about the ligands that are present in the PDB entry and every ligand in this category is assigned a alphanumeric binding site identifier. The _struct_site_gen category contains information of the residues that are present within the vicinity of the ligands described in the struct_site category. Both the categories are joined by the binding site identifier.

The site-delineation software used in PDB annotation is derived from the CCP4 (15) program CONTACT (http://www.ccp4.ac.uk/html/contact.html). It determines which polymeric residues make up the binding site for each non-polymeric ligand present in the structure (Figure 5), using a cut-off distance of 3.7 Å from any atom of the ligand and taking crystallographic symmetry into account. For oligosaccharides and peptide-like inhibitors and antibiotics the software generates binding-site information for the entire molecule instead of for the individual moieties (Figure 6). In addition to the software-generated site information, any author-provided details about catalytic site residues in a protein are also included in the annotated entry. In the mmCIF file for a given entry, the CIF categories *_struct_site* and *_struct_site_gen* contain the site information for all the ligands present in the structure. The *struct_site* category contains residue-level information. Each binding site is identified using a unique alphanumeric identifier (AC1, AC2, … , ZZ9) which serves as the primary key for the *_struct_site *category. The *_struct_site_gen *category contains information about all the neighbouring residues within 3.7 Å of any atom of the ligand. The data item *_struct_site_ gen.site_id* is directly inherited from the *struct_site.id* of the *_struct_site* category (Figure 7). In addition to the binding-site annotation, all covalent bonds between ligands and polymeric residues or other ligands are annotated as covalent linkages in the *_struct_conn* category of the PDBx/mmCIF file.

**Figure 8.**
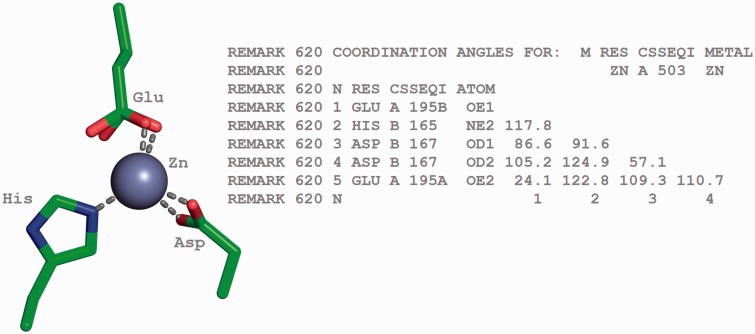
Tetrahedrally coordinated Zn ion in entry 2VW4 (32) along with the annotation of the bond angles. The REMARK 620 annotation indicates the software calculated bond angle values between Zn A 503 and its surrounding residues. The surrounding residues in anticlockwise direction are Glu A 195, HisB 165 and Asp B 167. The sidechain carboxylate group of the Glu residue exists in two alternate conformation (A and B conformers). The angle between GluA195B-Zn-HisB165 is 117.8, GluA195B-Zn-Asp(OD1)B167 is 86.6, HisB165-Zn-Asp(OD1)B167 is 91.6, Glu195B-Zn-ASP(OD2)B167 is 105.2, HisB165-Zn-Asp(OD2)B167 is 124.9, Asp(OD1)B167-Zn-Asp(OD2)B167 is 57.1, Glu(OE1)A195B-Zn-Glu(OE2)A195A is 24.1, HisB165-Zn- Glu(OE2)A195A is 122.8, Asp(OD1)B167-Zn-Glu(OE2)A195A is 109.3 and Asp(OD2)B167-Zn-Glu(OE2)A195A is 110.7.

In protein and DNA structures, metal ions are often found in coordination complexes with other molecules or ions. The metals or metal clusters present in metalloproteins, such as haemoglobins, transferrins, cytochromes, nitrogenases and hydrogenases are bound to nitrogen, sulphur or oxygen atoms belonging to protein residues. The details of the bond angles for each of these coordinated metal centres are listed in REMARK 620 of the PDB file (Figure 8) or the *_pdbx_struct_conn_angle* category of the PDBx/mmCIF file.

## Annotation of peptide-like inhibitors and antibiotics

In 2011, a major remediation was carried out by wwPDB curators to improve the representation of peptide-like inhibitors and antibiotics so that they can be more easily identified and studied (16). Currently, there are nearly 1300 such structures present in the PDB archive.

The presence or absence of consecutive peptide bonds determines how these molecules are represented in the PDB. If a peptide-like molecule contains two or more consecutive peptide bonds, it is annotated as a standard polymer with information about recommended name, source organism and linkage information between the amino acids and any non-standard residues present in the molecule. Cross-referencing to UniProt (17) for gene products or to Norine (18) for non-ribosomal peptides is attempted for such compounds.

If the ligand has fewer than two consecutive peptide bonds (examples of this include several PPACK inhibitors and other peptide-like molecules), it is annotated as a non-polymer like any other small molecule in the CCD, but with an identifiable subcomponent sequence running from amino terminal (N) to carboxy (C) terminal.

**Figure 9.**
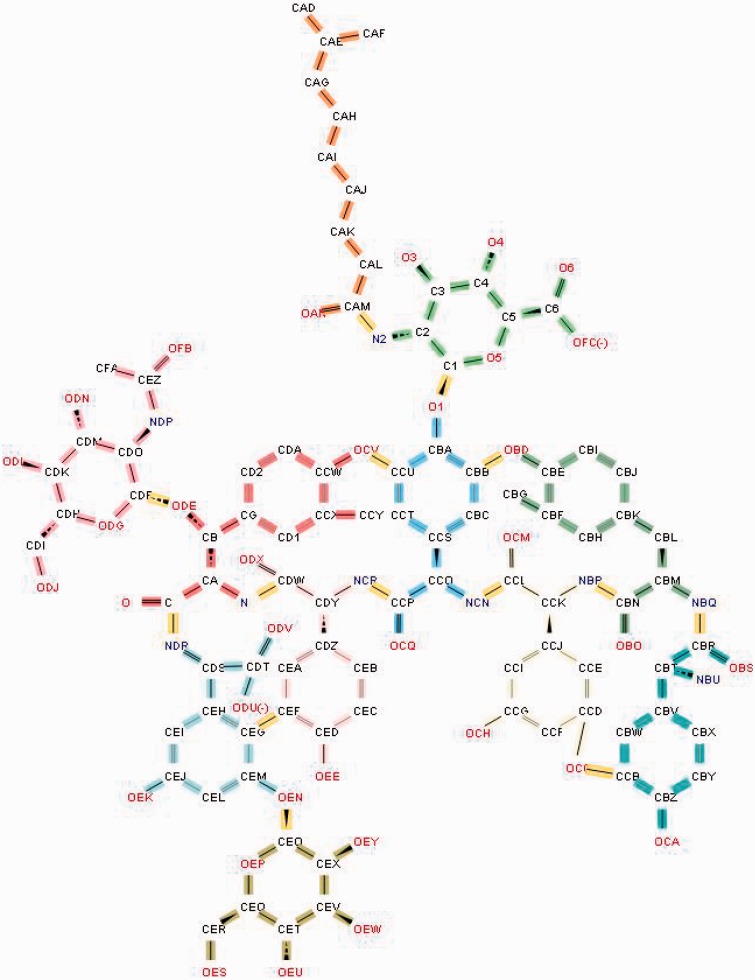
Annotation of the glycopeptide antibiotic teicoplanin involves ‘chopping up’ the molecule into its component chemical entities that are validated against the CCD. The bonds highlighted in yellow demarcate the individual entities.

Antibiotics such as teicoplanin and vancomycin have sugars or fatty acids attached to their peptide core. Such compounds are represented as a ‘group’ (16), which includes the polymeric and non-polymeric constituents of the antibiotic along with details about all the linkages between them (Figure 9).

## BIRD

The large amount of chemical and biological information that emerged during the remediation of the peptide-like antibiotic and inhibitor molecules in the PDB required systematic referencing and documenting for curation of future depositions. A new resource was created to assist the future annotation of such compounds, containing both chemical and biological information. The chemical information is stored in Peptide Reference Dictionary (PRD) files, whereas the biological function is documented in a separate family file. The chemical and biological information are separately annotated so that chemically similar molecules, either with conserved core polymer sequence or signature sequence motifs, can be grouped under the same family of antibiotics or peptide-like molecules. For example, antibiotics, such as chlorionectin A, vancomycin, teicoplanin and balhimycin are part of the glycopeptide antibiotic family. For each chemically distinct peptide-like molecule a new PRD description is created with a unique identifier and information about its chemical composition, connectivity, structural description (e.g. glycopeptide, anthracycline, lipopeptide, etc.) and function (e.g. immunosuppressant, enzyme inhibitor, thrombin inhibitor, etc.). Information about a family of molecules is stored in a FAM file, which mainly includes biological annotation (such as function, mechanism of action and pharmacological action) and the list of family members in the PRD (16). Remediated and newly released PDB entries containing peptide-like compounds include PRD identifiers in the PDBx files. The new resource, comprising both chemical and biological information for these peptide-like molecules, is called the BIRD. Both the PRD and family files are distributed via the wwPDB ftp area (ftp://ftp.wwpdb.org/pub/pdb/data/bird). Currently, there are nearly 200 families and more than 700 PRD entries in BIRD. The release of any new PRD entries or families is synchronized with the release of the first PDB entry containing the corresponding peptide-like molecule.

## Validation of ligands in the PDB

**Figure 10.**
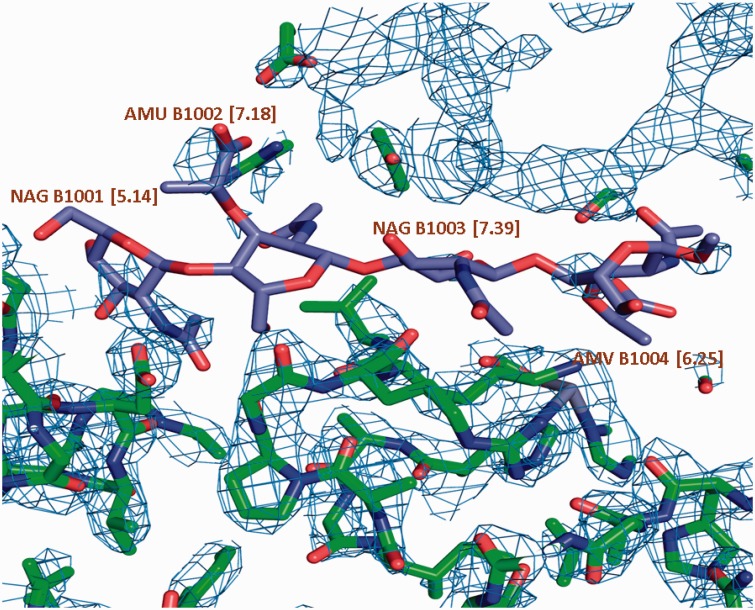
σ_A_ weighted 2Fo-Fc map of a carbohydrate binding protein shown at a contour level of 0.35e/A^3. Very little electron density is observed for the oligosaccharide molecule. This is reflected in the high LLDF values (shown in parentheses) for each of the component carbohydrate moieties.

The wwPDB structure validation process includes geometry checks for all standard and non-standard amino acids and nucleotides as well as carbohydrates and ligands. Since 2008, wwPDB has convened several method-specific validation task forces (VTFs), composed of community experts, to provide recommendations on structure and data validation (19–21). The recommendations of the X-ray VTF have been implemented in a software pipeline that is used during deposition and annotation, as a stand-alone server, and for validation of all legacy crystal structures in the archive (22). The validation pipeline uses a variety of external and internal software. To assess bond lengths, bond angles, torsion angles and chirality for every small molecule present in an entry, use is made of the program Mogul (23), which compares the geometry to that encountered in high-quality small molecule structures in the CSD (24). The validation pipeline also analyses the fit of ligands to the electron density using approaches introduced by the Uppsala Electron-Density Server (25). For every ligand in an entry, the local ligand density fit (LLDF) is calculated; this is essentially a *Z*-score of the ligand’s real space *R*-value (RSR) (26) relative to the mean and standard deviation of the RSR values of the neighbouring polymeric standard residues and nucleotides (taking crystallographic symmetry into account), and using a cut-off distance of 5 Å from any ligand atom. LLDF values >2 are highlighted in the report produced by the validation pipeline (Figure 10). The validation report generated during the annotation process is sent back to the depositor so it can be included when a paper describing the structure is submitted for publication. Upon release of an entry, the validation pipeline is run on it again, and the resulting report is made publicly available.

## Conclusions and future directions

The PDB archive contains a wide variety of chemical entities, many of which play a crucial role in modulating biochemical reactions. Correct identification and representation of these small molecules in the PDB at the annotation stage is crucial to allow these molecules to be identified, compared, etc. by users. Every structure deposited to the PDB is dissected to identify the individual chemical entities that are subsequently compared and validated against the CCD. When a chemical entity matches an existing dictionary entry, it is assigned the same three-character identifier as in the CCD. In addition, the nomenclature of the entity’s atoms is updated to reflect the dictionary definition. Once the individual components have been correctly identified, information is added about all the non-standard covalent, H-bonding and ionic interactions that occur in the structure. The annotation software also generates binding-site information for every bound molecule. For all crystal structures, symmetry information is taken into account during the identification of binding sites and non-covalent interactions.

The rapid growth and increased complexity of the structures to PDB has prompted continuous review of the annotation tools and processes used during processing and handling the data deposited to the PDB. To support the scientific advancements in the field of structural biology, the wwPDB partners are collaborating to develop a new joint deposition and annotation system (27). The new annotation system contains four major modules, such as a chemical component module, a sequence module, a validation module and a module for derived data. The chemistry module can perform simultaneous searches against both the CCD and BIRD. The new system has an interactive interface and visualization tools that aid in annotation and facilitate correct representation of the data (28). The chemistry module has been tested on previous depositions and has significantly improved processing efficiency and data quality.
